# TSG101 and PEG10 are prognostic markers in squamous cell/adenosquamous carcinomas and adenocarcinoma of the gallbladder

**DOI:** 10.3892/ol.2014.1886

**Published:** 2014-02-14

**Authors:** ZIRU LIU, ZHULIN YANG, DONGCAI LIU, DAIQIANG LI, QIONG ZOU, YUAN YUAN, JINGHE LI, LUFENG LIANG, MEIGUI CHEN, SENLIN CHEN

**Affiliations:** 1 Research Laboratory of Hepatobiliary Diseases, Second Xiangya Hospital, Central South University, Changsha, Hunan 410011, P.R. China; 2 Department of Pathology, Second Xiangya Hospital, Central South University, Changsha, Hunan 410011, P.R. China; 3 Department of Pathology, Third Xiangya Hospital, Central South University, Changsha, Hunan 410013, P.R. China; 4 Department of Pathology, Basic School of Medicine, Central South University, Changsha, Hunan 410078, P.R. China; 5 Department of Hepatobiliary and Pancreatic Surgery, Hunan Provincial People’s Hospital, Changsha, Hunan 410007, P.R. China; 6 Department of Pathology, Loudi Central Hospital, Loudi, Hunan 417011, P.R. China; 7 Department of Pathology, Hunan Provincial Tumor Hospital, Changsha, Hunan 410013, P.R. China

**Keywords:** gallbladder cancer, adenocarcinoma, squamous cell carcinoma, adenosquamous carcinoma, TSG101, PEG10, prognosis, metastasis

## Abstract

The clinicopathological characteristics of squamous cell/adenosquamous carcinoma (SC/ASC) are currently not well documented, and as the prevalence of SC/ASC is uncommon in gallbladder cancers, a prognostic marker has not yet been found. In the present study, the expression of tumor susceptibility gene (TSG) 101 and paternally expressed gene (PEG) 10 was assessed in 46 SC/ASCs and 80 adenocarcinomas (ACs) using immunohistochemistry, and the samples were further analyzed to examine correlations with the clinicopathological characteristics. It was demonstrated that positive TSG101 and PEG10 expression were significantly associated with large tumor size, high tumor-node-metastasis (TNM) stage, lymph node metastasis, invasion and no resection (only biopsy) of SC/ASC and AC. The univariate Kaplan-Meier analysis showed that positive TSG101 and PEG10 expression, and differentiation, tumor size, TNM stage, lymph node metastasis, invasion and surgical curability, is closely associated with a decreased overall survival in SC/ASC and AC patients (P<0.05 or P<0.001). The multivariate Cox regression analysis identified that positive TSG101 and PEG10 expression are independent factors for a poor-prognosis in SC/ASC and AC patients. The present study indicates that positive TSG101 and PEG10 expression are closely associated with the clinical, pathological and biological behaviors, and a poor prognosis in gallbladder cancer.

## Introduction

In the USA, gallbladder cancers (GBCs) are the most common biliary tract malignancy and the fifth most common gastrointestinal cancer (1,2). The prognosis of GBC is extremely poor, with a high mortality rate, and early diagnosis is generally impossible due to a lack of specific signs or symptoms (3). The majority of GBC patients (>90%) are diagnosed at an inoperable stage, with serious invasion and metastasis to other organs (4). The majority of GBCs are adenocarcinomas (ACs; >90%) (5). By contrast, it is rare for other histopathological subtypes, including mucinous, papillary and squamous subtypes, to be identified (2). Between 1 and 12% of gallbladder cancers are squamous cell/adenosquamous carcinomas (SC/ASCs) (2,6), and the clinicopathological characteristics of SC/ASCs are not well documented, as the majority of available studies are individual case studies or analyses of small case series. The establishment of therapeutic interventions for SC/ASC is required (2). Currently, biomarkers for predicting the prognosis of AC are under investigation, however, none have achieved clinical application as of yet (4). Notably, biomarkers associated with the progression and prognosis of SC/ASC have not been reported, and therefore, documenting the clinicopathological and biological characteristics is essential.

Paternally expressed gene (PEG) 10 was first identified by Ono *et al* as an imprinted gene that is paternally expressed and maternally silenced (8). The human PEG10 gene is located on chromosome band 7q21, functioning as a transcriptional factor. PEG10 expression can be detected in a variety of human normal tissues, including the brain, kidney, lung, placenta, testis, ovary, spleen, lymphoblasts, endothelial cells and thymus (9,10), however, its exact roles remain unknown. The overexpression of the PEG10 gene has also been detected in human cancers, including leukemia, breast cancer, hepatocellular carcinoma (HCC), prostate cancer and pancreatic cancer (7,11). The exact association between PEG10 and tumorigenesis has not yet been identified. However, the accumulated evidence indicates the involvement of PEG10 in apoptotic resistance and oncogenesis. For instance, in studies of B-cell acute lymphoblastic leukemia, PEG10 mRNA expression was strongly associated with high lipoprotein lipase expression, which is a predictor of unfavorable outcome in B-cell chronic lymphocytic leukemia (10), whereas overexpressed PEG10 increased apoptotic resistance in B cell lineage, acute and chronic lymphocytic leukemia cluster of differentiation (CD)23^+^/CD5^+^ B cells (12). In HCC, PEG10 decreases cell death through interaction with seven in absentia homolog-1, a mediator of apoptosis (9). Previous studies have demonstrated that PEG10 expression can be regulated by the proto-oncogene, c-MYC, via the binding of the c-MYC oncoprotein to the E-box-containing region of the first intron of PEG10 (13–15). PEG10 also interacts with the transforming growth factor (TGF)-β type I receptor, activin receptor-like kinase (ALK) 1 (16). Additionally, knockdown of PEG10 inhibits the proliferation of pancreatic carcinoma and HepG2 HCC cells (13), while knockout of the PEG gene causes early embryonic lethality (17). This evidence indicates that PEG10 may play a crucial role in carcinogenesis and tumor cell growth. However, PEG10 expression in SC/ASC and AC of the gallbladder has not yet been identified.

Although tumor susceptibility gene (TSG) 101 was originally identified as a potential tumor suppressor gene (18), subsequent studies have shown that the deletion of TSG101 in cell cultures did not lead to uncontrolled cell growth, while conditional knockout of TSG101 in mice did not result in neoplastic transformation. However, homozygous deletion of TSG101 led to embryonic lethality in gene knockout mice, whereas cell cycle arrest and cell death resulted from the silencing of TSG101 expression in mammalian cells (18). This indicates that TSG101 plays a crucial role in cell survival. In addition, a previous study has indicated that TSG101 is an essential protein involved in numerous cellular processes associated with cell growth and signal transduction, including transcriptional regulation, protein ubiquitination, cell cycle control and vesicular transport (20). Liu *et al* reported overexpression of TSG101 in human papillary thyroid carcinomas, which provided one of the earliest pieces of evidence for linking PSG101 to carcinogenesis (21). The overexpression of TSG101 was also observed in several human cancers, including ovarian cancer (19), gastrointestinal tumors (22) and colorectal carcinoma (23). Gene silencing of TSG101 leads to growth arrest and cell death in breast and prostate cancer cells (24). In addition, early evidence indicated the close interaction of TSG101 with p53 within the p53/mouse double minute (MDM) 2 homolog feedback control loop, which upon de-regulation, results in tumorigenesis (25). However, no studies have shown the involvement of TSG101 in gallbladder cancer.

In the present study, the expression of PEG10 and TSG101 in resection specimens, including 80 AC and 46 SC/ASC samples, were examined by immunohistochemistry. The correlations of PEG10 and TSG101 expression with the biological behavior and prognosis of SC/ASC and AC of the gallbladder were evaluated, along with the clinical significance and the survival rates of the patients.

## Materials and methods

### Case selection

Between January 1995 and December 2009, 46 SC/ASC samples were collected from patients who had undergone surgical resection or biopsy. In the gallbladder cancers of the present study, the percentage of SC/ASC was 4.34% (46/1,060 GBCs). Among the 46 SCs/ASCs, 14 samples (14/325 GBCs) were collected from Xiangya Hospital, 16 (16/370 GBCs) from Second Xiangya Hospital, 5 (5/110 GBCs) from Third Xiangya Hospital, 5 (5/105 GBCs) from Hunan Provincial People’s Hospital, 4 (4/100 GBCs) from Hunan Provincial Tumor Hospital (all Changsha, Hunan, China) and 1 each from Changde Central Hospital and Loudi Central Hospital (Loudi, Hunan, China), respectively (2/50 GBCs). Between January 2001 to December 2009, a total of 80 AC samples from patients who had undergone surgical resection or biopsy, were collected from Second Xiangya Hospital and Loudi Central Hospital. This study was approved by the Ethics Committee for Human Research, Central South University (Changsha, China).

In total, there were 27 female and 19 male (F/M, 1.42) SC/ASC patients, and 54 female and 26 male (F/M, 2.08) AC patients. The age range was 35–82 years (mean ± SD, 55.8±9.6 years) for the SC/ASC patients and 33–80 years (mean ± SD, 53.8±9.9 years) for the AC patients. The differentiation classifications of the squamous cells of the SCs/ASCs samples included 16 well-differentiated (34.8%), 24 moderately-differentiated (52.2%) and 6 poorly-differentiated (13.0%) carcinomas. For the AC samples, 27 samples were well-differentiated (33.8%), 25 were moderately-differentiated (31.3%) and 28 were poorly-differentiated (35.0%). Invasion of the gallbladder, surrounding tissues and organs was identified in 30 SC/ASC patients (65.2%), while 29 had regional lymph node metastasis (63.0%) and 28 had gallstones (60.9%). Invasion was found in 49 AC patients (61.3%), while 50 had regional lymph node metastasis (62.5%) and 38 had gallstones (47.5%). According to the tumor-node-metastasis (TNM) staging, 5 of the SC/ASC samples were stage I tumors, 7 were stage II, 20 were stage III and 14 were stage IV. For the AC samples, 8 were stage I tumors, 13 were stage II, 38 were stage III and 21 were stage IV. In total, for the SCs/ASCs and ACs, 14 and 26 patients underwent radical resection surgery, 18 and 28 underwent palliative surgery and 14 and 26 underwent no operation and only had biopsies, respectively.

The 2-year survival data of the SC/ASC and AC patients was collected from phone calls and letters. In total, 23 AC patients survived >1 year (9 patients survived >2 years) and 57 survived <1 year, with an average survival time of 10.34±0.63 months. Among the SCs/ASCs patients, 13 survived >1 year (4 patients survived >2 years) and 33 survived <1 year, with an average survival time of 10.07±0.78 months.

### Immunohistochemistry staining

Sections (4-μm thick) were cut from routinely paraffin-embedded tissues of AC and SC/ASC. Rabbit anti-PEG10 and mouse anti-TSG101 antibodies were purchased from Santa Cruz Biotechnology, Inc. (Santa Cruz, CA, USA). The staining was performed with the peroxidase-based EnVision™ Detection kit (Dako Laboratories, Carpinteria, CA, USA), following the manufacturer’s instructions. In brief, 4-μM sections were cut from routinely paraffin-embedded tissues of the AC and SC/ASC samples. The sections were soaked with phosphate-buffered saline (PBS) for 3×5 min prior to the sections being deparaffinized and incubated with 3% H_2_O_2_ for 15 min. The sections were then incubated with mouse anti-TSG101 (1:100 dilution) or rabbit anti-PEG10 (1:100 dilution) antibody for 1 h at room temperature. Solution A (containing horseradish peroxidase-conjugated secondary antibody) was added subsequent to the rinsing of the sections with PBS (3 times), and then the sections were incubated for 30 min. The substrate, 3,3′-diaminobenzidine, was added prior to hematoxylin counter-staining. Following dehydration, the slides were soaked in xylene 3 times, for 5 min each. For the positive control, positive sections were purchased from Foochow Maixin Biotechnology Company (Foochow, China), and for the negative control, the primary antibody was replaced with 5% fetal bovine serum. The percentage of positive cells was calculated from 500 cells in 10 random fields; ≥25% positive cells were regarded as positive and <25% positive cells were regarded as negative.

### Statistical analysis

The data were analyzed using the statistical package for the Social Sciences, Version 13.0 (SPSS, 13.0; SPSS, Inc., Chicago, IL, USA). The inter-association of TSG101 or PEG10 expression with histological or clinical factors was analyzed using χ^2^ or Fisher’s exact tests. Kaplan-Meier and time series (log-rank) tests were used for the univariate survival analysis. Cox’s proportional hazards model was used for the multivariate analysis and to determine the 95% confidence interval. P<0.05 was used to indicate a statistically significant difference.

## Results

### Comparison of TSG101 and PEG10 expression and clinicopathological characteristics in SC/ASC and AC

**Figure 1:**
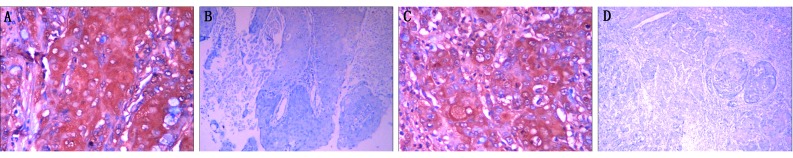
TSG101 and PEG10 expression in SC/ASC using EnVision immunohistochemistry; original magnification, ×200. TSG101- and PEG10-positive reactions were mainly localized in the cytoplasm. (A) Positive TSG101 expression (>25% positive cells) in moderately-differentiated SC/ASC. (B) Negative TSG101 expression (<25% positive cells) in well-differentiated SC/ASC. (C) Positive PEG10 expression in poorly-differentiated SC/ASC. (D) Negative PEG10 expression in moderately-differentiated SC/ASC. TSG101, tumor susceptibility gene 101; PEG10, paternally expressed gene 10; SC/ASC, squamous cell/adenosquamous carcinoma.

**Figure 2:**
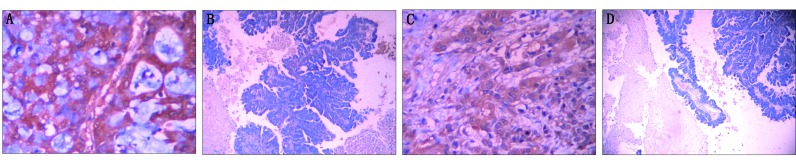
TSG101 and PEG10 expression in AC using EnVision immunohistochemistry; original magnification, ×200. TSG101- and PEG10-positive reactions were mainly localized in the cytoplasm. (A) Positive TSG101 expression (>25% positive cells) in poorly-differentiated AC. (B) Negative TSG101 expression (<25% positive cells) in well-differentiated AC. (C) Positive PEG10 expression in poorly-differentiated AC. (D) Negative PEG10 expression in well-differentiated AC. TSG101, tumor susceptibility gene 101; PEG10, paternally expressed gene 10; AC, adenocarcinoma.

As shown in Table I, the percentage of cases with a patient age of >45 years, a tumor mass of >3 cm and well- or moderately-differentiated tumors was significantly higher in the SCs/ASCs compared with the ACs (P<0.05). Correlations between other clinicopathological characteristics and the percentage of positive TSG101 and PEG10 expression were not significant. The majority of TSG101- and PEG10-positive reactions were localized in the cytoplasm of the SC/ASCs (Fig. 1) and ACs (Fig. 2), as observed using EnVision immunohistochemistry (Dako Laboratories).

### Association of clinicopathological characteristics and TSG101 and PEG10 expression in SC/ASC and AC patients

A significantly higher association was apparent between the percentage of cases with TSG101- and PEG10-positive expression in the SC/ASC samples with a large tumor mass size, high TNM stage, lymph node metastasis, invasion and no resection (biopsy only) compared with the cases of small tumor size, low TNM stage, no lymph metastasis, no invasion and radical resection (P<0.05; Table II).

For AC tumors, the percentage of TSG101- and PEG10-positive expression was significantly higher in the cases with poor differentiation, large tumor mass size, high TNM stage, lymph node metastasis, invasion and collection of tumor samples by biopsy, compared with the well-differentiated cases, small tumor mass, low TNM stage, no lymph node metastasis, no invasion and collection of tumor samples by resection (P<0.05 or P<0.01; Table III).

### Correlation between survival rates and TSG101 or PEG10 expression in patients with SC/ASC and AC

The survival information of the SC/ASC and AC patients was collated from phone calls and letters. The follow-up time for the present study was 2 years. The patients with a survival time >2 years were included as censored cases in the analysis. In total, 57 AC patients survived >1 year and 23 survived <1 year (9 survived >2 years), with an average survival time of 10.34±0.63 months. For the SC/ASC patients, 33 survived <1 year and 13 survived >1 year (4 survived >2 years), with an average survival time of 10.07±0.78 months.

**Figure 3:**
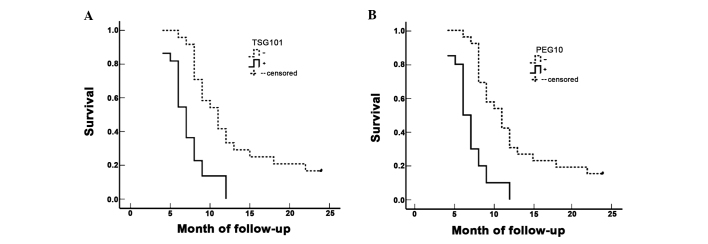
TSG101 and PEG10 expression and survival in patients with SC/ASC of the gallbladder. (A) Kaplan-Meier plots of overall survival time in patients with SC/ASC and with positive and negative TSG101 expression. (B) Kaplan-Meier plots of overall survival time in patients with SC/ASC and with positive and negative PEG10 expression. TSG101, tumor susceptibility gene 101; PEG10, parentally expressed gene 10; SC/ASC, squamous cell/adenosquamous carcinoma.

Evaluation of the SC/ASC patients using a Kaplan-Meier survival analysis demonstrated that differentiation, tumor size, TNM stage, lymph node metastasis, invasion and surgical procedure (P<0.001) were significantly associated with average survival time (Table IV), and the average survival time of the TSG101- and PEG10-positive patients was significantly lower than that of the patients with a negative result for TSG101 and PEG10 expression (P<0.001; Table IV and Fig. 3). Cox’s multivariate analysis demonstrated that the differentiation, tumor size (≥3 cm), TNM stage, invasion, surgical procedure and TSG101- and PEG10-positive expression were negatively correlated with overall survival, indicating that the positive expression of TSG101 and PEG10 is a risk factor of SCs/ASCs (Table V).

**Figure 4:**
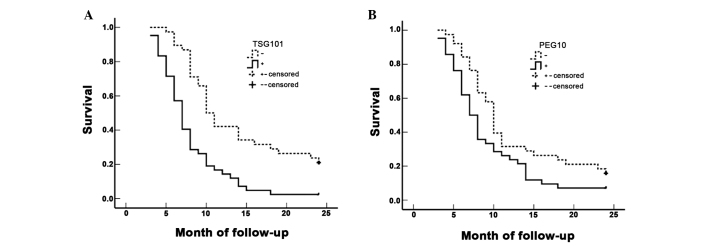
TSG101 and PEG10 expression and survival in patients with AC of the gallbladder. (A) Kaplan-Meier plots of overall survival time in patients with AC and with positive and negative TSG101 expression. (B) Kaplan-Meier plots of overall survival time in patients with AC and with positive and negative PEG10 expression. TSG101, tumor susceptibility gene 101; PEG10, parentally expressed gene 10; AC, adenocarcinoma.

The Kaplan-Meier survival analysis of the AC patients revealed similar results as for the SC/ASC patients (Table VI). The average survival time of the TSG101- or PEG10-positive AC patients was significantly lower than patients exhibiting negative TSG101 or PEG10 expression (P<0.001; Table VI and Fig. 4). Cox’s multivariate analysis demonstrated that differentiation, tumor size (≥3 cm), TNM stage, lymph node metastasis, invasion, surgical procedure and TSG101- and PEG10-positive expression positively correlated with the poor survival rate of the AC patients (Table VII).

## Discussion

The current knowledge on the clinicopathological characteristics of SC/ASC has mainly been obtained from individual case studies or analyses of small case series. Therefore, accurate understanding of the differences between rare SC/ASC tumors and ordinary adenocarcinomas is not possible without further studies. The reported incidence of squamous differentiation is 1–12% in gallbladder malignancies (26,27), and in the present study 4.34% SCs/ASCs were observed. A previous study identified that the occurrence of SC/ASC is predominant in females (F/M, 3.8) (25), however in the present study there was no significant difference (F/M, 1.4). It was also apparent in the present study that the prevalence of SC/ASC was more significant in older patients compared with AC. In previous studies, it has been demonstrated that the proliferation of SC occurs at a higher rate than AC, whereas the prevalence of squamous tumors is less frequent with lymph node metastasis (28,29). Observations from the present study revealed no differences in the occurrence of invasion and lymph node metastasis between AC and SC/ASC, however, more SC/ASC patients had a larger tumor size. In total, 86% of SC/ASC and 74% of AC patients were diagnosed at an inoperable stage, however, for the remaining patients it was apparent that radical resection was a good prognostic factor for AC and SC/ASC. There was no significant difference in the post-operative survival time between cases of AC (10.34±0.63 months) and SC/ASC (10.07±0.78 months). Furthermore, no significant differences in differentiation, TNM stage and surgical curability were found between AC and SC/ASC. These observations indicated that the clinicopathological presentations of SC/ASC did not appear to be significantly different from ordinary AC, and that squamous differentiation was no more aggressive than glandular differentiation in the gallbladder.

A previous study has demonstrated that the PEG10 gene is overexpressed in leukemia, breast cancer, prostate cancer, hepatocellular carcinoma and pancreatic cancer (11). Knockdown of PEG10 has been shown to inhibit the proliferation of cancer cells (13). Further evidence has demonstrated that PEG10 expression can be regulated by the proto-oncogene, MYC (4), and that PEG10 also interacts with the TGF-β type I receptor, ALK1 (16). Notably, the expression of human telomerase reverse transcriptase is downregulated when PEG10 is knocked down by siRNA (15). This evidence indicates the involvement of PEG10 in carcinogenesis. Similarly, the overexpression of TSG101 has been detected in human papillary thyroid carcinomas (21), ovarian cancer (19), gastrointestinal tumors (22) and colorectal carcinoma (23). Silencing of TSG101 leads to growth arrest and cell death in breast and prostate cancer cells (24). Additionally, overexpression of TSG101 plays an oncogenic role by inactivating p53 through MDM2 upregulation (21,25). This evidence also strongly indicates that TSG101 is involved in tumorigenesis. Certain studies have found that TSG101 is overexpressed in the vincristine-resistant human gastric adenocarcinoma cell line, SGC7901/VCR (30). The same group later found that the silencing of TSG101 expression significantly increased SGC7901/VCR sensitization to chemotherapeutic drugs through reducing adverse drug reactions (31), indicating that TSG101 plays a critical role in chemoresistance. Although no studies have revealed that PEG10 is directly involved in chemoresistance, overexpressed PEG10 is involved in apoptotic resistance (12). This may be an explanation for why chemotherapy and radiation therapy exhibit less of an effect in GBC.

Although the overexpression of PEG10 and TSG101 in cancer cells has been previously studied, their expression in SC/ASC and AC of the gallbladder has yet to be identified. In the present study, an extensive collection of human SC/ASC and AC samples was used to demonstrate that overexpressed PEG10 and TSG101 are associated with large tumor mass size, high TNM stage, lymph node metastasis, invasion and no resection (only biopsy) in SC/ASC and AC, and with poor differentiation in AC. It was also demonstrated that the survival time in patients with overexpression of PEG10 and TSG101 was significantly shorter when compared with patients with lower expression; Cox’s multivariate analysis indicated that the overexpression of PEG10 and TSG101 was positively correlated with mortality. The present study indicates that the function of PEG10 and TSG101 may be involved in the progression, metastasis and prognosis of AC and SC/ASC.

In conclusion, the elevated expression of PEG10 and TSG101 in gallbladder SC/ASC and AC samples indicates that they are significant markers for progression, clinical biological behavior and prognosis. The involvement of TSG101 in chemoresistance and the role of PEG10 in apoptosis resistance indicate that these two markers have a strong potential to be developed as a target for gene therapy, which may sensitize chemotherapy and radiotherapy. Also, patients with high PEG10 and TSG101 expression in their tumors are more likely to suffer from invasion and metastatic recurrence. These patients may require close monitoring for clinical signs of relapse, so that therapeutic inventions can be applied early enough for optimal outcomes.

## Figures and Tables

**Table I:** Comparison of gallbladder SC/ASC and AC clinicopathological features and TSG101 and PEG10 expression status.

Clinicopathological characteristics	SC/ASC (n=46)	AC (n=80)	χ^2^	P-value
Gender, n (%)				
Male	19 (41.3)	26 (32.5)	0.986	0.352
Female	27 (58.7)	54 (67.5)		
Age, n (%)				
≤45 years	3 (6.5)	16 (20.0)	4.143	0.042
>45 years	43 (93.5)	64 (80.0)		
Differentiation, n (%)				
Well	16 (34.8)	27 (33.8)		
Moderate	24 (52.2)	25 (31.3)	8.515	0.014
Poor	6 (13.0)	28 (35.0)		
Maximum tumor diameter, n (%)				
≤3 cm	20 (43.5)	50 (62.5)	4.280	0.039
>3 cm	26 (56.5)	30 (37.5)		
Cholecystolithiasis, n (%)				
(−)	18 (39.1)	42 (52.5)	2.093	0.148
(+)	28 (60.9)	38 (47.5)		
TNM stages, n (%)				
I+II	12 (26.1)	21 (26.3)		
III	20 (43.5)	38 (47.5)	0.287	0.866
IV	14 (30.4)	21 (26.3)		
Lymph node metastasis, n (%)				
(−)	17 (37.0)	30 (37.5)	0.004	0.952
(+)	29 (63.0)	50 (62.5)		
Locoregional invasion, n (%)				
(−)	16 (34.8)	31 (38.8)	0.197	0.658
(+)	30 (65.2)	49 (61.3)		
Surgical methods, n (%)				
Radical	14 (30.4)	26 (32.5)		
Palliative	18 (39.1)	28 (35.0)	0.215	0.898
Without resection	14 (30.4)	26 (32.5)		
Mean survival time, months (range)	10.07 (4–25)	10.34 (3–27)	0.014	0.906
TSG101, n (%)				
(−)	24 (52.2)	38 (47.5)	0.951	0.382
(+)	22 (47.8)	42 (52.5)		
PEG10, n (%)				
(−)	26 (56.5)	38 (47.5)	0.289	0.678
(+)	20 (43.5)	42 (52.5)		

TSG101, tumor susceptibility gene 101; PEG10, paternally expressed gene 10; SC/ASC squamous cell/adenosquamous carcinoma; AC, adenocarcinoma; TNM, tumor-node-metastasis.

**Table II:** Association of TSG101 and PEG10 expression with the clinicopathological characteristics of SC/ASC.

Clinicopathological characteristics	Cases, n	TSG101			PEG10		
	
		Pos, n (%)	χ^2^	P-value	Pos, n (%)	χ^2^	P-value
Gender							
Male	19	8 (42.1)	0.425	0.515	7 (36.8)	0.580	0.446
Female	27	14 (51.9)			13 (48.1)		
Age							
≤45 years	3	1 (33.3)	0.270	0.603	1 (33.3)	0.134	0.714
>45 years	43	21 (48.8)			19 (44.2)		
Pathological type							
SC	26	14 (53.8)	0.869	0.351	14 (53.8)	2.616	0.106
ASC	20	8 (40.0)			6 (30.0)		
Differentiation							
Well	16	6 (37.5)	3.753	0.153	6 (37.5)	1.573	0.456
Moderate	24	11 (45.8)			10 (41.7)		
Poor	6	5 (83.3)			4 (66.7)		
Tumor mass size							
≤3 cm	20	6 (30.0)	4.506	0.032	5 (25.0)	4.916	0.028
>3 cm	26	16 (61.5)			15 (57.7)		
Gallstones							
No	18	9 (50.0)	0.056	0.813	10 (55.6)	1.755	0.185
Yes	28	13 (46.4)			10 (35.7)		
TNM stage							
I+II	12	3 (25.0)			2 (16.7)		
III	20	8 (40.0)	8.282	0.017	8 (40.0)	8.059	0.018
IV	14	11 (78.6)			10 (71.4)		
Lymph metastasis							
No	17	4 (23.5)	6.379	0.012	4 (23.5)	4.367	0.037
Yes	29	18 (62.1)			16 (55.2)		
Invasion							
No	16	4 (25.0)	5.123	0.024	3 (18.8)	6.105	0.016
Yes	30	18 (60.0)			17 (56.7)		
Surgery							
Radical	14	3 (21.4)	9.296	0.010	3 (21.4)	7.374	0.025
Palliative	18	8 (44.4)			7 (38.9)		
Biopsy	14	11 (78.6)			10 (71.4)		

TNM, tumor-node-metastasis; TSG101, tumor susceptibility gene 101; PEG10, paternally expressed gene 10; Pos, positive; SC/ASC, squamous cell/adenosquamous carcinoma.

**Table III:** Association of TSG101 and PEG10 expression with the clinicopathological characteristics of AC.

Clinicopathological characteristics	Cases, n	TSG101			PEG10		
	
		Pos, n (%)	χ^2^	P-value	Pos, n (%)	χ^2^	P-value
Gender							
Male	26	13 (50.0)	0.097	0.756	13 (50.0)	0.097	0.756
Female	54	29 (53.7)			29 (53.7)		
Age							
≤45 years	16	6 (37.5)	1.805	0.179	5 (31.3)	3.622	0.057
>45 years	64	36 (56.3)			37 (57.8)		
Differentiation							
Well	27	9 (33.3)	9.865	0.007	10 (37.0)	6.800	0.034
Moderate	25	12 (48.0)			12 (48.0)		
Poor	28	21 (75.0)			20 (71.4)		
Tumor mass size							
≤3 cm	50	21 (42.0)	5.896	0.015	22 (44.0)	3.863	0.049
>3 cm	30	21 (70.0)			20 (66.7)		
Gallstones							
No	42	22 (52.4)	0.001	0.982	20 (47.6)	0.845	0.358
Yes	38	20 (52.6)			22 (57.9)		
TNM stage							
I+II	21	5 (23.8)			6 (28.6)		
III	38	20 (52.6)	13.749	0.001	19 (50.0)	11.736	0.003
IV	21	17 (81.0)			17 (81.0)		
Lymph metastasis							
No	30	9 (30.0)	9.744	0.002	11 (36.7)	4.825	0.032
Yes	50	33 (66.0)			31 (62.0)		
Invasion							
No	31	11 (35.5)	5.877	0.015	12 (38.7)	3.860	0.049
Yes	49	31 (63.3)			30 (61.2)		
Surgery							
Radical	26	7 (26.9)	13.052	0.001	9 (34.6)	9.282	0.010
Palliative	28	15 (53.6)			14 (50.0)		
Biopsy	26	20 (76.9)			19 (73.1)		

TNM, tumor-node-metastasis; TSG101, tumor susceptibility gene 101; PEG10, paternally expressed gene 10; AC, adenocarcinoma; Pos, positive.

**Table IV:** Association between TSG101 and PEG10 expression, clinicopathological characteristics and average survival of SC/ASC patients.

Clinicopathological characteristics	Cases, n	Average survival, months (range)	χ^2^	P-value
Gender				
Male	19	10.74 (6–24)	0.767	0.381
Female	27	9.85 (4–24)		
Age				
≤45 years	3	15.67 (8–24)	2.023	0.155
>45 years	43	9.84 (4–25)		
Pathological type				
SC	26	10.19 (4–24)	0.223	0.637
ASC	20	10.25 (4–24)		
Differentiation				
Well	16	13.81 (5–24)		
Moderate	24	8.92 (4–18)	19.125	<0.0001
Poor	6	5.83 (4–9)		
Tumor mass size				
≤3 cm	20	14.35 (7–24)	31.337	<0.0001
>3 cm	26	7.04 (4–11)		
Gallstones				
No	18	8.22 (4–12)	3.730	<0.0001
Yes	28	11.50 (4–24)		
TNM stage				
I+II	12	17.00 (9–24)		
III	20	9.20 (7–15)	51.139	<0.0001
IV	14	5.86 (4–8)		
Lymph node metastasis				
No	17	14.24 (4–24)	16.219	<0.0001
Yes	29	7.86 (4–15)		
Invasion				
No	16	15.75 (9–24)	32.271	<0.0001
Yes	30	7.27 (4–12)		
Surgery				
Radical	14	16.64 (10–24)		
Palliative	18	8.50 (6–12)	50.165	<0.0001
Biopsy	14	6.00 (4–8)		
TSG101				
−	24	12.96 (6–24)	16.277	<0.0001
+	22	7.23 (4–12)		
PEG10				
−	26	12.73 (6–24)	19.275	<0.0001
+	20	6.95 (4–12)		

TSG101, tumor susceptibility gene 101; PEG10, paternally expressed gene 10; SC/ASC squamous cell/adenosquamous carcinoma; TNM, tumor-node-metastasis.

**Table V:** Multivariate Cox regression analysis of survival rate in SC/ASC patients.

							95% confidence interval	
							
Groups	Factors	RC	SE	Wald	P-value	RR	Lower	Upper
Pathological types	SC/ASC	0.189	0.363	0.271	0.603	1.208	0.593	2.461
Differentiation	Well/Moderate/Poor	1.167	0.402	8.427	0.004	3.212	1.461	7.063
Tumor mass size	≤3 cm/>3 cm	2.343	0.777	9.093	0.003	10.412	2.271	47.747
Gallstone	No/Yes	1.018	0.521	3.818	0.051	2.768	0.997	7.684
TNM stage	I+II/III/IV	1.170	0.517	5.121	0.024	3.222	1.170	8.876
Lymph metastasis	No/Yes	1.061	0.421	6.351	0.012	2.889	1.266	6.594
Invasion	No/Yes	2.389	0.785	9.262	0.002	10.903	2.341	50.785
Surgery	Radical/Palliative/Biopsy	1.068	0.487	4.809	0.028	2.910	1.120	7.557
TSG101	−/+	1.126	0.491	5.259	0.022	3.083	1.178	8.072
PEG10	−/+	1.194	0.486	6.036	0.014	3.300	1.273	8.555

RC, Regression coefficients; SE, standard error; RR, relative risk; TNM, tumor-node-metastasis; TSG101, tumor susceptibility gene 101; PEG10, parentally expressed gene 10; SC/ASC, squamous cell/adenosquamous carcinoma.

**Table VI:** Association between TSG101 and PEG10 expression, clinicopathological characteristics and average survival time of AC patients.

Clinicopathological characteristics	Cases, n	Average survival, months (range)	χ^2^	P-value
Gender				
Male	26	9.58 (3–24)	2.567	0.109
Female	54	11.30 (3–24)		
Age				
≤45 years	16	10.81 (4–24)	0.003	0.956
>45 years	64	10.72 (3–24)		
Differentiation				
Well	27	15.07 (5–24)		
Moderate	25	10.60 (4–24)	32.501	<0.0001
Poor	28	6.68 (3–14)		
Tumor mass size				
≤3 cm	50	13.70 (6–24)	68.283	<0.0001
>3 cm	30	5.80 (3–10)		
Gallstones				
No	42	10.19 (3–24)	0.246	0.620
Yes	38	11.34 (4–24)		
TNM stage				
I+II	21	18.96 (5–24)		
III	38	9.29 (6–15)	105.825	<0.0001
IV	21	5.14 (3–7)		
Lymph node metastasis				
No	30	16.27 (4–24)	42.372	<0.0001
Yes	50	7.42 (3–14)		
Invasion				
No	31	16.68 (7–24)	55.535	<0.0001
Yes	49	6.98 (3–11)		
Surgery				
Radical	26	18.31 (10–24)		
Palliative	28	8.64 (6–11)	113.141	<0.0001
Biopsy	26	5.42 (3–9)		
TSG101				
−	38	13.76 (5–24)	18.937	<0.0001
+	42	8.00 (3–24)		
PEG10				
−	38	12.40 (4–24)	4.677	0.031
+	42	9.24 (3–24)		

TNM, tumor-node-metastasis; TSG101, tumor susceptibility gene 101; PEG10, paternally expressed gene 10; AC, adenocarcinoma.

**Table VII:** Multivariate Cox regression analysis of survival rate in AC patients.

							95% confidence interval	
							
Groups	Factors	RC	SE	Wald	P-value	R	R Lower	Upper
Differentiation	Well/Moderate/Poor	1.192	0.449	7.048	0.008	3.294	1.366	7.941
Tumor mass size	≤3 cm/>3 cm	1.127	0.430	6.869	0.009	3.086	1.329	7.169
Gallstone	No/Yes	0.213	0.262	0.661	0.416	1.237	0.740	2.068
TNM stage	I+II/III/IV	1.282	0.452	8.045	0.005	3.604	1.486	8.740
Lymph metastasis	No/Yes	1.456	0.548	7.059	0.008	4.289	1.465	12.555
Invasion	No/Yes	1.420	0.501	8.033	0.005	4.137	1.550	11.045
Surgery	Radical/Palliative/Biopsy	1.420	0.468	9.206	0.002	4.137	1.653	10.353
TSG101	−/+	1.198	0.480	6.229	0.013	3.313	1.293	8.489
PEG10	−/+	1.140	0.495	5.304	0.021	3.127	1.185	8.250

a RC, Regression coefficients; SE, standard error; RR, relative risk; TNM, tumor-node-metastasis; TSG101, tumor susceptibility gene 101; PEG10, parentally expressed gene 10; AC, adenocarcinoma.
